# Government and public health responses to e-cigarettes in New Zealand: vapers’ perspectives

**DOI:** 10.1186/s12954-018-0219-9

**Published:** 2018-04-05

**Authors:** Trish Fraser, Marewa Glover, Penelope Truman

**Affiliations:** 1 Global Public Health, P O Box 82, Glenorchy, 9350 New Zealand; 2 School of Health Sciences, College of Health, Massey University, Private Box 756, Wellington, 6140 New Zealand

**Keywords:** E-cigarettes, Nicotine, Vaping

## Abstract

**Background:**

The New Zealand (NZ) government is to lift the ban on the sale of nicotine for use in electronic cigarettes (e-cigarettes).

**Methods:**

Using a naturalistic approach, we sought to understand how the current law was experienced by e-cigarette users (vapers). Twenty-nine vapers were interviewed by telephone, between May and September 2016, using a semi-structured interview schedule. Open-ended questions covered: initiating vaping, the experience of stopping smoking, technical problems encountered, reasons for vaping, acceptability of vaping, addiction to vaping and advice given to smokers about vaping. The audio recordings were transcribed and then independently coded using a general inductive thematic analysis.

**Results:**

This paper presents the main theme which was that vapers employed a range of reactionary strategies to the ban on the sale of nicotine e-liquid in NZ. These included lobbying government, spreading the word, establishing vaper support groups, helping people stop smoking by switching to vaping and advocating for e-cigarettes to be incorporated into smoking cessation practice.

**Conclusions:**

Vapers’ experience and observations form a popular or lay epidemiology––one that identified that e-cigarettes were helping people stop smoking and could thus deliver public health benefits. Public health researchers and workers, and government fears about vaping, and proposals to strengthen restrictions contributed to the growth of the vaper community who reacted by forming self-help groups and providing alternative cessation support to smokers. For a significant switch from smoking to vaping to occur, the health sector needs to have a change of attitude towards vaping that is positive, and the public needs evidence-based information on vaping. A first step could be for the health sector to collaborate with the vaping community to reorient current tobacco control and cessation practice to encourage smokers to switch to less harmful smoke-free alternatives to smoking.

## Background

Smoking and other high-risk ways of using tobacco are causally associated with the death of about 6 million people each year [1]. In 2013, the World Health Assembly established a global voluntary tobacco target to reduce the prevalence of tobacco use by 30% in people aged 15 years and over [1]; however, the prevalence of smoking is still increasing globally [2]. New Zealand with its 2025 goal of 5% or below smoking prevalence goes much further than the World Health Assembly goal but is on track to achieve this only for the most privileged [3].

Nicotine containing electronic cigarettes (e-cigarettes) as the biggest ever competitor to smoked tobacco [4] may be one solution to reducing inequities in smoking prevalence. The growth of the use of e-cigarettes (vaping) as a social practice [5] has challenged conventional narratives about nicotine and tobacco, sparking a divisive debate among public health researchers and advocates [2]. Concerns include that vaping may have health risks [6] and may lead to renormalisation of smoking [7], increased uptake of smoking [8], increased nicotine addiction [9] and a reduction in quitting smoking [10]. Counter arguments are that e-cigarettes pose lower risks to individuals than smoking tobacco [11, 12], help smokers quit [13, 14], are generally not used by non-smokers [15], and vapers are thought to have a lower dependency on e-cigarettes than smokers have on cigarettes [16]. Debate continues over whether experimentation with e-cigarettes is associated with smoking initiation. While some studies find no ‘gateway’ effect [17] or perverse effects, such as restrictions on vaping being associated with smoking among adolescents [18], many studies find the opposite [19]. Additionally, vaping has some positive effects, such as reversing smoking-related harm [20] and potentially modelling stop smoking behaviour [21].

Many countries have responded negatively to e-cigarettes. As in October 2016, 25 countries had confirmed existing restrictions on nicotine would be applied to nicotine containing vaping products or new legislation to ban the sale of e-cigarettes was introduced [22]. In November 2016, the Framework Convention on Tobacco Control (FCTC) 7th Conference of the Parties (COP7) recommended that countries ban or regulate the importation, sale and distribution of e-cigarettes [23]. The NZ government had already applied an existing Smoke-free Environments Act 1990 (SFEA) clause banning the import of *oral* tobacco products for sale and distribution to nicotine containing e-cigarettes and e-liquid [24]. Just prior to COP7, however, the government announced that the SFEA would be amended to reverse the ban on sales of nicotine for vaping, and regulations would be introduced to govern marketing, access to and the quality of vaping products [25]. In summary, by mid to late 2018, vaping products would be able to be sold wherever smoking tobacco products are sold; sales to under 18-year-olds, advertising (except point-of-sale advertising) and vaping in workplaces and other places where smoking is not allowed under the SFEA would be prohibited; and product safety requirements would be established, for example, for nicotine concentration [25]. In a surprising move, the NZ government followed this up with plans to establish a pre-market approval process for any other existing or new products established to be harm-reduced alternatives to smoking tobacco [26]. The government's rationale was that they were ‘focused on health outcomes and ensuring there were options’, regardless of who manufactured the product, that would support people to stop smoking [27]. A change of government in late 2017 has cast some doubt over the speed with which the changes might occur [28].

Since the commercial launch of the first e-cigarette a decade ago, the prevalence of vaping has continued to rise globally. It is estimated that 3.7% of adults vape in the United States of America (US) [29], 4% in the United Kingdom (UK) [2] and 2% in Europe [30]. Despite the barriers to accessing nicotine for vaping, preliminary data suggests 1.5% of adults in NZ vape currently which included daily to less than monthly [31]. For context, in NZ, 16% of adults aged 15 and over smoke currently which included at least monthly smoking, though there are large disparities by socio-economic status and ethnicity [32].

Support for vaping in NZ has been mixed but is changing. For example, the Ministry of Health’s position in March 2017 was that there was insufficient evidence ‘to recommend e-cigarettes confidently as a smoking cessation tool’. In October 2017, they announced their increased focus ‘on harm reduction with an aim to support smokers to switch to significantly less harmful products like e-cigarettes’ and they said stop smoking services should support smokers who wanted to use e-cigarettes within a quit attempt [33]. This aligned with the pre-existing lay public’s view. In a survey of NZ smokers, one third believed e-cigarettes could help people to stop smoking, 41% considered it acceptable to use them as a replacement product and 58% as a stop smoking aid [34]. There had also been considerable interest in e-cigarettes as a stop smoking aid among high prevalence groups such as low socio-economic smokers [35] and Maori and Pacific smokers [36].

Much of the research on e-cigarettes and vaping reflects expert views and has been focused on identifying and quantifying the risks of vaping and vaping products including whether they will help or hinder smoking cessation [37]. Studies by, with or even on, vapers are currently rare. In a few surveys, vapers have reported their concerns that e-cigarette regulations will be overly restrictive [38, 39]. A survey of Australian vapers found that participants were suspicious of the government in relation to e-cigarettes [5]. Australia classifies liquid nicotine as a controlled poison which means people caught with nicotine containing vaping products who do not have a Doctor’s prescription can be fined and imprisoned under respective State laws. Most States and Territories have also applied their existing extensive smoking bans to vaping [22]. This highly restrictive response to e-cigarettes was seen by some survey participants as unethical and counterproductive to the goal of reducing harm from smoking [5].

### The importance of lay epidemiology

Just as there has been a call for research that prioritises smokers’ own perspectives [40] so too has there been a call for more naturalistic research on vapers’ perspectives of vaping [5]. The knowledge and experience of people who have vaped represents a large, international and growing lay epidemiology. Lay, or popular, epidemiology describes the public’s interpretation and understanding of health risks [41] and their participation in public policy, advocacy, and the pursuit of scientific knowledge [42, 43]. Lay epidemiology often precedes and prompts scientific awareness and involvement [42]. Brown [42] observed a pattern of stages through which a lay epidemiology emerges starting with an affected community noticing unexpected health effects. With regard to vaping, it became noticeable in the early years after the launch of e-cigarettes that people were stopping smoking and not relapsing as often as had been commonly experienced previously. The rest of Brown’s lay epidemiology development stages appear salient for investigating the rise of vapers as a group producing a body of lay knowledge. For instance, in the second stage, the community hypothesise a causal link to explain the effect they have observed and they begin to share their stories with each other creating a common perspective (Brown’s stage three). With the formation of a more cohesive group, stage four is initiated leading to a more concerted gathering of anecdotal ‘evidence’ and attempts to talk with researchers, health professionals and government officials about their knowledge and experience. If they are dismissed and ignored, typically, this fuels the establishment of advocacy groups and alliances (stage five) who attract researchers to conduct studies (stage seven). The building evidence is then used to confront opposition and if necessary pursue litigation (stage eight). Stage six, which chronologically occurs around this point, is that well-funded government and academic scientific studies begin to be conducted. Meanwhile, the affected community and their advocacy groups press for corroboration of their findings and official recognition (stage nine).

Lay epidemiology plays an important, if sometimes unwelcome role, in the ‘consensual production’ of scientific knowledge [42, 44]. It is often unwelcome when it is in opposition to public health which especially occurs if public health messages are false or exaggerated [41] as they sometimes have been regarding the risks of e-cigarettes, vapour and nicotine [45]. Lay epidemiology can help explain why people do not change their behaviour in the prescribed way, if at all [44], or, for instance, why smokers continue to try to quit unassisted rather than using funded cessation support and subsidised cessation medicines [46]. Vapers are the real experts argue Allmark and Tod [41]. They are best placed to help ‘outsiders’ [47], especially those of us who have never smoked or vaped, to understand why e-cigarettes are now the preferred method for stopping smoking [48].

Recognising the influence of lay knowledge on health behaviours is critical to developing acceptable and effective public health policy and interventions [49]. At the time of the development of the Ottawa Charter for Health Promotion, the WHO stated that ‘more emphasis should be laid on qualitative methods of observation, namely, those that allow lay people to define a problem and its solution from their viewpoint’ [50].

Using a naturalistic approach, this study aimed to understand how the current NZ law prohibiting the import, sale and distribution of nicotine for vaping was perceived by vapers. At the time of the study, the government had not announced a clear position either way on strengthening restrictions or legalising the sale of nicotine containing e-liquids.

## Methods

A qualitative exploratory method using in-depth interviews was chosen given that vaping is a relatively new phenomenon. Participants were drawn from a larger online survey of NZ vapers conducted to determine patterns of vaping in NZ, explained more fully in Truman, Glover & Fraser [51]. A purposive sub-group of survey participants was selected to enhance the analysis of the survey data and provide a deeper understanding of how, when and why vapers vape; how easy it is to vape and to buy e-cigarettes; how useful e-cigarettes are as stop smoking aids; whether e-cigarettes reduce craving for nicotine; whether vaping brings any benefits or risks; and how family, friends and others respond to vaping. We also asked participants for their views on what vaping means for them and the experience of vaping in a country without local legal access to nicotine for vaping. They were also invited to add information they deemed important to the topic beyond what we had asked. This paper reports on vapers’ response to regulations on the sale and purchase of vaping products, particularly nicotine for e-cigarettes. Themes on switching from smoking to vaping and perceived addictiveness of smoking versus vaping are presented elsewhere (Glover, Truman, and Fraser, in preparation).

### Participants

Of the 218 participants in our broader internet-based survey of NZ resident vapers [51], 72 provided a telephone number and consent to participate in an in-depth interview. Thirty of these participants were randomly selected and mapped against a purposive sampling frame (non-random targeting of online survey participants with specific characteristics) to ensure a demographically diverse range of people with different vaping characteristics were selected. Criteria for this sampling frame were females as well as males (at least 5 of each category), a mix of ethnicities including Māori and Pacific people (at least 3 of each category), and vapers who only vaped as well as those who vaped and smoked (at least 5 of each category). To fulfil these criteria, we tried to contact the third Pacific participant who had provided a telephone number, but he did not respond to efforts to contact him. As only 4 vapers who vaped and smoked had provided telephone numbers, these 2 groups were both under-represented.

### Recruitment

Thirty-three participants in total were contacted during May to September 2016 until 30 people who met our criteria had consented or saturation was reached (no more new information was forthcoming). Potential participants were sent a text or telephoned and invited to participate in a telephone interview lasting approximately 20 min and at a time convenient to them ensuring that we got as much information as they had to give [52].

### Interview process

A semi-structured interview schedule was used to guide the interviews, which were recorded on an i-Phone and a backup recording device. The interview schedule contained six domains of open-ended questions: initiating vaping and experience of stopping smoking if applicable, technical problems encountered, reasons for vaping, perceived acceptability of vaping, perceived addiction to vaping and advice they would give to smokers about vaping. The interviewers (TF, MG) asked open-ended questions and allowed participants to talk freely. Prompts were used to facilitate a naturalistic flow of related questions in the interview schedule or to seek a more in-depth explanation. Clarification was sought if information was provided that the interviewer did not understand, for example, unique vernacular that has developed around vaping or the equipment used. At the end of the interview, the interviewer checked that all the question realms had been covered. Participants were asked if they had anything to add. Interviews ranged in duration from 17 to 50 min.

### Data analysis

The audio recordings were transcribed verbatim. Each transcript was read independently by two of the authors (TF, MG) who identified distinct meaning units in the content which were then assigned a category label. A general inductive approach was used to allow new or unexpected categories to emerge from the data [53]. The two researchers then compared their coding, discussed category labels and discrepancies. Where necessary, discrepancies in coding were discussed until a consensus was reached [54]. Categories were grouped into over-arching themes for description.

### Ethics

In the current contested e-cigarette climate, we recognise that vapers, as research participants, are vulnerable and there is a growing distrust among vapers towards researchers and a suspicion that their views will be misrepresented [55]. To mitigate misrepresenting vapers or vaping, we consulted two experienced vapers on the larger online survey design and questions. We also attended ‘vape days’ to gain a better understanding of vapers and their views.

Ethical approval for the study was obtained from the Ministry of Health, Health and Disability Ethics Committee (Ethics Number 15/STH/215).

## Results

Of the 29 participants interviewed, the majority were NZ European males, between the age of 41 and 50 years old, who only vaped (did not smoke). The characteristics of the participants are summarised in Table 1.

**Table 1:** Characteristics of participants

Number	
Age	
20–30	4
31–40	5
41–50	14
51–60	3
61–70	3
Gender	
Male	21
Female	8
Ethnicity (multiple responses)	
NZ European	20
Māori	6
Pacific	2
Other	2
Vaped and/or smoked	
Only vaped	25
Vaped and smoked	4

A major theme to emerge from the study was that NZ vapers felt that they had been abandoned by the health sector and that their continued access to vaping products was threatened. Vapers’ reactions to this threat and dismissal of their concerns when raised are presented in this paper.

Vapers expressed disappointment that the health sector had left them largely to fend for themselves. The government and health sector’s behaviour had shocked participants. Some sensed an injustice perpetrated against them as vapers, and against smokers who might have quit smoking if vaping had been supported. In the vacuum left by the health sector, vapers had taken on the role of public health advocates and were implementing a range of change strategies. Five categories of actions were described: (1) lobbying local and central government, (2) spreading the word, (3) establishing vaper support groups, (4) helping smokers switch to e-cigarettes and (5) advocating for vaping to be supported by the health system. Further, some felt compelled to act in these ways, for instance to become politically active by advocating and lobbying against anti-vaping sentiment, sourcing information about vaping products, finding and analysing research on e-cigarettes and vaping, and seeking and extending support to other vapers. Individually and in groups, some participants voluntarily began to help smokers switch to vaping, a task they believed government funded stop smoking services should have been doing. Though frustrated at the position they had been put in by the government, they also expressed immense pride individually for having quit smoking and immense pride in the vaping community for the integrity and generosity perceived to epitomise their newfound community.

### Lobbying government (national and local)

Overwhelmingly, the major concern voiced by participants was that nicotine for vaping was incorrectly, they believed, classified as illegal and that nicotine e-liquids and e-cigarettes were inappropriately inaccessible or burdensome to obtain. They talked about lobbying politicians for local availability of nicotine for vaping. A couple of participants reported lobbying their local member of parliament (MP) to make nicotine more accessible for smokers wanting to quit. One participant said he sends information to his MP on a regular basis. Several participants were involved in lobbying politicians and making submissions on e-cigarettes.

I have told [MP] it’s [the nicotine e-cigarette] worked and I have sent him shit-loads of emails ‘cause he said to me ‘look if you get any information about whether it’s positive or negative can you let me know?’ ...So, if I see anything now, any studies or anything like that I just fire them off to him cause he wanted to have a good look at it. Whether that’s just the politician talking, he seemed pretty serious about it. (Male, NZ Eu, 41–50, only vaped).

Actually, my niece got me to write a letter to one of the Ministers the other day about my e-cig journey. She sent me an email address and said ‘Aunty can you write a letter to the Minister and tell them about what the e-cig has done for you and how you’ve used yours?’ (Female, Māori, 41–45, only vaped).

Several vapers had lobbied their local Councillors to try and prevent the inclusion of vaping in outdoor smoking bans. Participants from Wellington mentioned that the Wellington City Council had banned vaping in outdoor areas where smoking had also been banned. One female participant, who advocated that vaping not be included in the ban, said ‘Wellington City Council wanted to ban it all. I’ve written to them…’. She said, she challenges the ban by ‘having the odd vape’ as she walks through Civic Square in Wellington, which is close to the Council buildings.

### Spreading the word

Most of the participants talked a lot about promoting vaping to smokers as a way to encourage them to quit smoking.

…been doing quite a lot in the scene as well, well not heaps but trying to get more people involved in looking at them [e-cigarettes] and taking them seriously. So definitely helping spread the word. (Male, NZ Eu, 26–30, smoked and vaped).

Often, they were motivated by their own success at quitting or the success of colleagues, friends and or family. A couple of participants had expected to be smokers for life and having quit by using e-cigarettes, they wanted to share their experience with others. Some participants referred to an e-cigarette as a ‘magic bullet’. The main method of promotion of e-cigarettes was distributing business cards of local vape shops and international websites where e-cigarettes containing nicotine and nicotine e-liquid could be purchased. One participant collected cards from websites she ordered nicotine from so she could pass them on to potential vapers. A couple of vapers actively promoted vaping widely to smokers to encourage them to switch to vaping, sometimes specifically vaping in smoking areas to act as a role model for vaping.

We always thought we were going to be stuck smoking cigarettes forever because that’s all we ever knew and we found something and we feel better and we are healthier and we want to share that and that’s something that a lot of vapers like to promote as well. (Female, Other, 41–50, only vaped).

I am a great advocate for it and if I can push it I will… People are always curious when I am out and I’ll give them a few puffs or let them try it but it normally knocks their socks off cause it’s a bit too tough on someone that’s just thinking about giving up cigarettes so I do not even do that anymore. (Male, NZ Eu, 61–65, only vaped).

### Establishing vaper support groups

Participants reported that vaping groups had been established by vapers interested in helping smokers’ transition from smoking to vaping.

We decided to do ‘Vape it Forward’, which is mentoring for people… we have loan equipment that we give to people and they get a person with that loan equipment, a mentor, and that person shows them how to use it [e-cigarette] and gets them going. (Female, Other, 41–50, only vaped).

Accompanying these vaping groups, participants described establishing or joining Facebook pages and fora which they said, not only was a way to provide advice and information to new vapers but also they were a place where vapers discussed a range of topics relating to vaping and vaping products. Some vapers described going to extraordinary lengths to help others, such as delivering nicotine e-liquid to fellow vapers who had posted on Facebook that they had run out. Participants believed that vapers can revert to smoking very easily if something happens to their e-cigarette or charger, or they run out of nicotine.

It’s amazing. The vaping community which is obviously a virtual community online or with Facebook groups or whatever, it’s hugely supportive. Like if you’ve got any troubles at all, someone will know the answer. You just put it out there and someone will be willing to help. So, there is just an amazing amount of collective knowledge that is very generously offered among the vaping community. People kind of see it as part of the role really, that you, get into it and you support others into it as well and figure it out together. (Female, NZ Eu, 46–50, only vaped).

### Helping smokers switch to e-cigarettes

Experienced vapers talked about giving smokers advice in the form of a referral to a local e-cigarette outlet or a website or assisting them throughout the process of purchasing an e-cigarette, as they perceived new vapers can get ‘overwhelmed’ by the choices available. Most vapers believed that without help many new vapers would relapse to smoking. In some cases, vapers said they lend, give or buy smokers e-cigarettes to get them started vaping.

I have given away about five of my early e-cigs to people and all five of them have stopped [smoking], only one of them went back… It’s an expensive one because I buy all the stuff then I give it away. The early ones I would rather give them away with a few free juices, which I normally get from my supplier in the States. Every time I order my juice they always send free samples and I just give them away when I give an e-cigarette away to get them [smokers] started. (Male, NZ Euro, 61–65, only vaped).

They were also often approached by smokers when they were vaping. This subsequently led to a discussion about e-cigarettes, or an offer to smokers to try vaping or to set them up with an e-cigarette. Most of the participants had ‘converted’ some smokers to vaping, usually colleagues, family and friends but sometimes strangers. A few believed they had helped many smokers switch to e-cigarettes in this way.

I’ve converted over 80 people, 60 of them are now vapers, the other 10 would be dual vaping and smoking. (Male, Māori, 41–50, only vaped).

Vapers often found themselves taking on the role of an e-cigarette expert recommending e-cigarettes to smokers as a ‘good tool’ to quit smoking. Key considerations taken into account when giving advice to potential vapers were budget, a person’s level of technical expertise and number of cigarettes smoked per day. Important factors participants identified for smokers transitioning from smoking to vaping successfully were nicotine strength, e-liquid flavour, cloud density and e-cigarette ease of use. According to most participants, once new vapers were confident with their initial e-cigarette setup, they would probably progress onto something a little more technical that would be likely to give them a better, more satisfying experience. Participants said that it was important for new vapers to have an understanding of the effectiveness of nicotine and how to access it. A couple of the participants spoke of not using nicotine initially because they did not know it was an essential part of vaping in order to quit smoking.

I’d advise them on a pen style vape with a good dose of nicotine depending on their habit. So, if they were a 20 a day person I’d put them on something like 12 mg, yeah make sure that they understand the nicotine laws. It’s a huge hassle. (Male, NZ Eu, 36–40, only vaped).

### Advocating for vaping to be incorporated into health systems

E-cigarettes had not generally been accepted by the health sector at the time of the study, and participants thought it was wrong that smokers wishing to use e-cigarettes to quit smoking were offered no guidance or support from public health or health professionals.

Some participants reported attempts to educate health promoters and stop smoking providers. They talked to their GPs and other stop smoking providers about e-cigarettes, and the success they had had with them and asked them why they did not offer advice on e-cigarettes.

I get a little bit frustrated when you go to the doctor ‘cause, they’ve got this nicotine replacement therapy (NRT) stuff that never ever worked for me. They can’t advocate or promote vaping. They don’t seem to want to know about it. (Female, NZ Eu, 56–60, only vaped).

The Quitcard [prescription for subsidised NRT] providers, they give out information and when I mention e-cigarettes it’s like ‘we can’t be saying it’s a therapeutic device and there’s not enough evidence’ and even some doctors, they’re on the fence about it. (Male, Māori, 41–50, only vaped).

Participants expressed uncertainty about where they could vape as there were no specific regulations for vaping, as there were for smoking, apart from a couple of Council policies. Some participants were annoyed as their perception was that they were expected to vape outside with smokers. They sometimes challenged expectations by vaping in smoke-free areas where they thought there would be a public expectation that vaping would not be allowed, for example, hospital grounds, supermarkets and areas where vaping has been specifically banned.

Some of the conversations I’ve had by the non-informed have been ‘well no you’re still smoking’. Now that was the Cancer Society in [town]. ‘No, you can’t do that here, it’s World Smokefree Day, that’s smoking’ and I said ‘actually, no it’s not. I am inhaling nicotine’. ‘Well, it looks like a cigarette. You need to go somewhere else please.’ (Female, Māori, 51–55, dual user).

## Discussion

Vapers, interviewed in this study, felt generally unsupported by the health sector at all levels, from the Ministry of Health to health professionals. The health sector was seen to be hindering them and smokers in general, from switching from smoking to vaping. As a result, NZ vapers had come to expect no help from health professionals. They reacted by developing their own and others’ expertise and knowledge of e-cigarettes. They formed as a community and lobbied for a law change to enable access to nicotine for vaping and the acceptance by health services of e-cigarettes as a stop smoking aid. They gathered and disseminated information to counter what they believed were exaggerations about the health risks of vaping and negative attitudes towards vapers, to create a supportive environment for smokers to switch to vaping. They established vaper support groups and fora, meeting online and in-person, and they directly assisted smokers to initiate and switch totally to vaping.

The chronology of actions reported in this study, that NZ vapers felt *compelled* to implement, mirrored Brown’s [42] stages of how lay epidemiology develops. Stage 1, of noticing that vaping helped participants to stop smoking where other methods had failed was evident to them. Sharing their stories with others and searching for research to confirm their experience mirrored stage 3, which led to communication and fora online and with doctors and MPs––stage 4. The rejection and hostility they encountered partly fuelled stage 5 activities such as the establishment of advocacy groups and vape community events. This paper documents the earlier stages of the development of the vaping-lay epidemiology in NZ. Later stages may follow. Indeed, this study focused on the vaping community’s experience, which could be said to be evidence of stage 6 activity in which government and academic studies begin to be conducted. It is hoped that stage 8 action such as pursuing litigation to facilitate recognition and support of the benefits of switching from smoking to vaping [56] will not be needed in NZ given the Ministry of Health’s support for vaping. The new government’s position on vaping is yet to become clear, leaving NZ vaping advocacy groups stuck repeating activity associated with stages 4 to 7, that is continuing to produce evidence and lobby for change.

Lay-health advocacy and social movements focused on improving health are not new and have been noted in other studies, such as those on toxic waste contamination [57] and AIDS [2]. Brown [42] goes as far as to say that health activism by those affected is necessary to make progress in health care and health policy. Lay epidemiology is more accurately a constant, although rarely acknowledged, contributor in the production of knowledge [42]. Highly divisive debates, such as those being raged over vaping, occur when a powerful orthodoxy feels threatened and moves to silence opposing views [42, 58]. The WHO’s FCTC-enshrined personal persuasion model for achieving ‘tobacco elimination’ [59] conflicts with a more negotiated [44] model that aims to empower people and develop community capacity to support change. The WHO quote above [50] urged a respect for lay people’s involvement, definition of the problem and identification of the solution. If left to develop their own solution to tobacco smoking, what would NZ smokers do? For most of our participants, smoking was an intractable problem they wanted to quit. NZ’s initial official response imposed barriers intended to deter smokers from switching to, what the vaping community believed, was the most effective harm-reduced alternative to smoking tobacco that had ever existed.

This study found that the NZ vaping community developed and has been providing an alternative ‘cessation service’ to smokers. Some participants helped smokers within their immediate work and social settings, sometimes loaning or providing equipment and e-liquid along with practical how-to guidance and follow-up to get people vaping. Others more proactively recruited smokers to ‘convert’ to vaping. Whatever way they did it, this alternative ‘cessation service’ was unfunded, based on peer support and genuine empathy, free to the recipient and convenient to them. Not only was this alternate ground-up ‘service’ cheaper, it is potentially more effective, and thus poses a real challenge to the government funded cessation services [14]. Russell et al. [60] concluded, based on their large international survey of vapers, that connecting people who smoked with former smokers who had switched to vaping represents a substantial opportunity to accelerate reduction in smoking prevalence. Vapers, they suggest, are likely to be more knowledgeable about how to use an e-cigarette to quit smoking, and they are enthusiastic about supporting others to do so.

In addition to the practical support to help smokers switch to vaping, many of our participants were concerned that the public had been misled about the risks of e-cigarettes. They reported that they and their community were engaged in combating this misinformation via their Facebook pages, vape-meets and personal conversations with family, people on the street, health professionals and MPs. A recent NZ survey confirmed misunderstanding about vaping is widespread––only 38% perceived vaping to be safer than smoking tobacco [31]. Misperceiving vaping to be as harmful as smoking tobacco could undermine the motivation to transition to vaping or worse - could cause vapers to stop vaping and return to smoking.

Embracing a harm reduction approach that supports smokers switching to significantly less harmful alternatives to smoking could assist NZ to meet its smoking prevalence goal of 5% or below by 2025. Bans on where people can vape, limiting nicotine concentration, and banning advertising (particularly internet advertising) could hinder smokers’ transitioning to vaping. These measures encourage health professionals to continue believing vaping should be discouraged. Further research is warranted to monitor changes in health professional’s perceptions of e-cigarettes and vaping and to investigate if vapers experience a supportive shift in public and professional attitudes towards their reduced harm behaviours once the law regulating vaping is passed.

Government and scientific opinion is based on evidence and international experience, but ‘popular’ or lay epidemiology is also influential [57]. When changes to the SFEA are made, it will be important that the benefits of vaping over smoking are optimised. For a significant public health benefit to be realised, the health sector will need to provide truthful evidence-based benefit and risk information and encouragement to switch to harm-reduced alternatives. A good first step would be for the health sector to learn from and, collaborate with, the vaping community to strategise how to encourage more smokers to switch to vaping. It may be, as Russell et al. [60] suggest, that effort should shift to supporting vaper-to-smoker peer led interactions in community contexts where these interactions are naturally occurring, for example, in vape shops, vape-meets and online fora. Further research is warranted to test the different switching support models that are emerging.

### Limitations

This was a small qualitative survey of NZ vapers who had participated in an online survey and had self-selected as being interested in this topic. Pacific people and vapers who also smoked were under-represented, but it may be that most people who vape do not smoke as well [51]. It is also likely that vapers who do not use websites or have contact with vape shops or the vaping community were unaware of the study and were therefore under-represented. The range of perspectives gathered may have been limited due to the polarised debate over e-cigarettes [58]. This is particularly so, Hoepner [58] warns, when ‘whole communities feel alienated by government or academic research institutions’ (pg 52) as our results suggest, this has been an experience for some vapers in NZ. Potential participants may also have been put off participating due to suspicion about the intent of the research [55] or to avoid feeling judged and stigmatised [49]. People who use e-cigarettes but who strongly reject the identity ‘vaper’ and people who had used e-cigarettes just to stop smoking would have been less likely to participate also [61]. A strength of the study was that experienced vapers were consulted on the design, and our research was grounded in the need to understand and listen to and give voice to vapers and their perception of the issues [2].

## Conclusions

Acknowledging the important contribution of lay epidemiology to the advancement of knowledge [42] and engaging in a proactive way with the people most affected [50] could have mitigated some of the hurtful impacts of the ‘irrational hostility and conflict’ [44] typical of protracted tribalised debates [58]. More importantly, smoking-related morbidity and mortality could have been reduced sooner and for more people.
